# A High-Density Genetic Map of Wild Emmer Wheat from the Karaca Dağ Region Provides New Evidence on the Structure and Evolution of Wheat Chromosomes

**DOI:** 10.3389/fpls.2017.01798

**Published:** 2017-10-20

**Authors:** Chad Jorgensen, Ming-Cheng Luo, Ramesh Ramasamy, Mathew Dawson, Bikram S. Gill, Abraham B. Korol, Assaf Distelfeld, Jan Dvorak

**Affiliations:** ^1^Department of Plant Sciences, University of California, Davis, Davis, CA, United States; ^2^Department of Statistics, University of California, Davis, Davis, CA, United States; ^3^Department of Plant Pathology, Kansas State University, Manhattan, KS, United States; ^4^The Institute of Evolution, University of Haifa, Haifa, Israel; ^5^Institute for Cereal Crops Improvement, George S. Wise Faculty of Life Sciences, Tel Aviv University, Tel Aviv, Israel

**Keywords:** inversion, single nucleotide polymorphism, SNP, translocation, *Triticum dicoccoides*, wheat evolution

## Abstract

Wild emmer (*Triticum turgidum* ssp. *dicoccoides*) is a progenitor of all cultivated wheat grown today. It has been hypothesized that emmer was domesticated in the Karaca Dağ region in southeastern Turkey. A total of 445 recombinant inbred lines of *T. turgidum* ssp. *durum* cv. ‘Langdon’ x wild emmer accession PI 428082 from this region was developed and genotyped with the Illumina 90K single nucleotide polymorphism Infinium assay. A genetic map comprising 2,650 segregating markers was constructed. The order of the segregating markers and an additional 8,264 co-segregating markers in the *Aegilops tauschii* reference genome sequence was used to compare synteny of the tetraploid wheat with the *Brachypodium distachyon*, rice, and sorghum. These comparisons revealed the presence of 15 structural chromosome rearrangements, in addition to the already known 4A-5A-7B rearrangements. The most common type was an intra-chromosomal translocation in which the translocated segment was short and was translocated only a short distance along the chromosome. A large reciprocal translocation, one small non-reciprocal translocation, and three large and one small paracentric inversions were also discovered. The use of inversions for a phylogeny reconstruction in the *Triticum–Aegilops* alliance was illustrated. The genetic map was inconsistent with the current model of evolution of the rearranged chromosomes 4A-5A-7B. Genetic diversity in the rearranged chromosome 4A showed that the rearrangements might have been contemporary with wild emmer speciation. A selective sweep was found in the centromeric region of chromosome 4A in Karaca Dağ wild emmer but not in 4A of *T. aestivum*. The absence of diversity from a large portion of chromosome 4A of wild emmer, believed to be ancestral to all domesticated wheat, is puzzling.

## Introduction

Wheat is the most widely grown food crop, and with rice and maize it plays the central role in the global food supply. Wheat species form a polyploid complex at three ploidy levels: diploid, tetraploid, and hexaploid. Two separate evolutionary lineages are recognized in this complex, but only the lineage that evolved from wild emmer, *Triticum turgidum* ssp. *dicoccoides* (genomes AABB), is economically important and will be considered here.

Wild emmer originated by hybridization of diploid *T. urartu* (genomes AA) with a species closely related to *Aegilops speltoides* (genomes SS, which are closely related to the BB genomes) (Dvorak and Zhang, 1990; Dvorak et al., 1993). Domestication of wild emmer in western Asia produced hulled domesticated emmer (*T. turgidum* ssp. *dicoccon*), which was an important crop in western Asia and northern Africa until it was replaced by free-threshing durum (*T. turgidum* ssp. *durum*) during the Greco-Roman times (Nesbitt and Samuel, 1996).

Today, the most important wheat is the hexaploid bread wheat, *T. aestivum* (genomes AABBDD). Bread wheat originated by hybridization of tetraploid wheat with diploid *A. tauschii* (genomes DD) (Kihara, 1944; McFadden and Sears, 1946). The tetraploid parent of bread wheat was likely a domesticated form of tetraploid wheat (Dvorak et al., 2012). Genetic evidence suggests that Caspian Iran (Wang et al., 2013) was the geographic place of bread wheat origin.

Wild emmer grows today in a discontinuous arc from Israel to western Iran and is subdivided into northern (Turkey, Iraq, and Iran) and southern (Israel, Lebanon, and southern Syria) populations (Ozkan et al., 2002; Luo et al., 2007). Plants of the northern population belong exclusively to the slender *horanum* race, but the southern population includes also a robust, *judaicum*, race. The latter may have originated by hybridization between wild emmer and durum (Blumler, 1997), although evidence for that is fragmentary (Luo et al., 2007).

Domestication of cereals, einkorn, emmer, and barley, was the hallmark of the emergence of agriculture in the Fertile Crescent (Harlan, 1975). Domesticated emmer began to appear in the southern Levant and southeastern Turkey about 10,000 years BP (Nesbitt and Samuel, 1996; Willcox, 1997). The initial studies based on amplified fragment length polymorphism (AFLP) or restriction fragment length polymorphism (RFLP) of nuclear DNA placed emmer domestication to the Karaca Dağ region in the northern portion of the Fertile Crescent (Nelson et al., 1995; Ozkan et al., 2002, 2005, 2011; Luo et al., 2007). Einkorn wheat was suggested to have been domesticated in the same area (Heun et al., 1997). Contradictory results were obtained in studies of organellar DNA, which placed emmer domestication in the northwestern portion of the Fertile Crescent in Turkey (Mori et al., 1997) or in southern Levant (Gornicki et al., 2014). Luo et al. (2007) suggested that emmer was domesticated in both the Karaca Dağ region and southern Levant. They suggested that the northern domesticated emmer population expanded and merged with emmer domesticated in the southern Levant. Another possibility is that emmer was domesticated in the southern Levant and the ‘wild’ emmer populations in the northern and eastern regions of the Fertile Crescent were actually populations of domesticated emmer that had become feral (Civan et al., 2013).

Wheat domestication was accompanied by selection for mutations in traits critical for wheat to function as a crop. The suite of these traits, such as non-brittle spike rachis, soft glume, large seed size, reduced tillering, erect growth habit, and others is called the domestication syndrome. The relationships among haplotypes of genes controlling these traits in a crop and its wild progenitor can provide valuable insights into the domestication process, its geography, and the subsequent evolution of the crop (Sweeney and McCouch, 2007).

Genetic dissection of crop domestication is predicated on the development of a mapping population for quantitative trait locus (QTL) mapping. A number of mapping populations from crosses between wild emmer and domesticated wheat have been reported (Peng et al., 2000; Peleg et al., 2005, 2008; Uauy et al., 2006; Avni et al., 2014; Faris et al., 2014a,b). Some of them have been used to map domestication genes (Peng et al., 2003; Distelfeld et al., 2007; Faris et al., 2014a,b; Tzarfati et al., 2014; Nave et al., 2016). However, none has involved wild emmer from what may be the most important region for the elucidation of emmer domestication, the Karaca Dağ region in southeastern Turkey.

Here, we report the development of a mapping population of recombinant inbred lines (RILs) from the cross of wild emmer accession PI 428082 from the Karaca Dağ region with durum cv. ‘Langdon’ and the use of this population in the construction of a genetic map needed for the study of the wheat domestication syndrome. Genetic studies suggested that durum is related to wild emmer in southern Levant (Luo et al., 2007), and prior information on gene sequences of Langdon and wild emmer from the Karaca Dağ region (Akhunov et al., 2010) indicated that there was sufficient polymorphism between Langdon and PI 428082 to construct a high-density genetic map from this cross. Langdon was chosen for this work because it has the “standard” wheat karyotype, a wealth of genetic stocks (Joppa et al., 1978), and a bacterial artificial chromosome (BAC) clone library (Cenci et al., 2003).

The construction of a high-density genetic map was facilitated by recent advances in mapping technology based on single nucleotide polymorphism (SNP) markers and Illumina genotyping platforms capable of massively parallel genotyping in the large and complex Triticeae genomes (Akhunov et al., 2009; Luo et al., 2013). The recently developed Illumina 90K wheat SNP Infinium assay (Wang et al., 2014) was employed for SNP genotyping of the RIL population. We also employed a recently completed genome sequence of *A. tauschii* (GenBank BioProject PRJNA341983) as a reference in ordering the co-segregating markers on the genetic map. That made it possible to align the genetic maps to the pseudomolecules of other grass genomes. These alignments were used to study the structure and evolution of wild emmer chromosomes, including the structurally rearranged chromosome 4A (Dvorak, 1983; Dvorak et al., 1990; Naranjo, 1992; Devos et al., 1995; Mickelson-Young et al., 1995; Nelson et al., 1995; Miftahudin et al., 2004; Hernandez et al., 2012; Balcárková et al., 2017).

## Materials and Methods

### Plant Material

Seeds of wild emmer accession PI 428082 were received from the US National Small Grains Collection, Aberdeen, Idaho. The accession was collected 52.2 km west of Diyarbakir (Latitude: 37° 46 min 59 s, Longitude: 39° 46 min 0 s) in the foothills (elevation 1400 m) of Karaca Dağ. The accession was crossed as the male parent with Langdon, which was received from L.R. Joppa (University of North Dakota, Fargo). Five F_1_ plants were self-pollinated to produce an F_2_ generation. The single seed descent technique was followed to advance generations by self-pollination in a greenhouse. A total of 445 independent F_6_–F_8_ RILs were developed.

### Genetic Map Construction

DNAs were isolated from leaf segments (Dvorak et al., 2006b) and genotyped using the wheat 90K iSelect Infinium genotyping assay (Illumina Incorporated, San Diego, CA, United States) at the UC Davis Genotyping Core facility. Output was analyzed with the GenomeStudio program (Illumina, San Diego, CA, United States). Genotype data were uploaded to the Mutipoint Ultra-dense (ULD) mapping program (MultiQTL Limited, Haifa, Israel) and processed as a RIL population. Only grouped (co-segregating) markers (Ronin et al., 2017) were employed in the first round of linkage map construction (marker “clustering”). This resulted in 55 linkage groups (LGs). Markers within a LG were ordered relative to each other using a “hard” setting of marker order monotony control. Marker order monotony was subsequently visually inspected, and markers that disturbed monotony were removed. In the next step, the ends of each LG were extended with singleton markers. The extended LGs that were within 0.1 recombination frequency (RF) were merged. Finally, singleton markers that showed linkage to internal marker groups in a LG were inserted.

The LGs were exported from Multipoint ULD to Microsoft Excel (Microsoft, Corp., Seattle, WA, United States) and compared to the 90K consensus map. This comparison was used to assign LGs to chromosomes, to determine their orientation, and to detect chimeric LGs. Observed RF values were then transformed into per-meiosis values (Haldane and Waddington, 1931) to compensate for the accumulation of recombination events across generations, from F_1_ to F_6_–F_8_. Lastly, centimorgan (cM) distances were computed from the transformed RF values using the Kosambi mapping function (Kosambi, 1943). The genetic map was compiled in Microsoft Excel, and a genotype matrix following the marker order on the genetic map was created. Graphical genotypes (Young and Tanksley, 1989) were scrutinized using a custom script using Python (Python Software Foundation, Beaverton, OR, United States) for inconsistent data points within the occasional heterozygous segments. The inconsistent data points were removed as part of quality control and all data points within a heterozygous block were labeled as missing data because the Multipoint ULD program could not be run in the specified mode with heterozygous data. This revised matrix was then used as the final input for the Multipoint ULD program to construct the final genetic map.

### Map Comparisons

The nucleotide sequences for the wheat 90K Illumina Infinium SNP markers were downloaded from the database (Wang et al., 2014) and used as BLASTN 2.2.28+ (NCBI) queries to search for homologous sequences in the *A. tauschii* pseudomolecules (GenBank BioProject PRJNA341983). A database was created, in which each SNP marker was associated with an *A. tauschii* subject hit. Colinearity between linkage map SNP markers and the *A. tauschii* pseudomolecules was assessed by searching for an ascending or descending order of top hit (subject) locations on the *A. tauschii* pseudomolecules. The arbitrary condition for declaring a marker colinear was that it was a member of a group of at least three different loci (genes) in a colinear order; otherwise markers were considered non-colinear.

Groups of colinear markers that indicated a chromosome rearrangement relative to *A. tauschii*, such as an inversion or translocation, were compared with consensus genetic maps of durum and common wheat (Maccaferri et al., 2014; Wang et al., 2014). Additional comparisons were made with the *Brachypodium distachyon*, v3.1 (Initiative International Brachypodium Genome, 2010), rice, v7.0 (Matsumoto et al., 2005), and sorghum v3.1 (Paterson et al., 2009) pseudomolecules to validate each structural change and to determine its ancestral versus derived state.

### Recombination Rate

Segregating markers were used to compute recombination rates, expressed as cM/Mb, using the cM position of a marker in the LG. Since a wheat genome sequence needed for the computation of recombination rates was not available to us we used the *A. tauschii* pseudomolecules (GenBank BioProject PRJNA341983) as the most closely related reference for the A and B genomes of tetraploid wheat. To estimate these rates, we employed local cubic kernel derivative smoothers with Gaussian kernel using the package KernSmooth in R (The R Foundation, Vienna Austria). The bandwidth used was 20 Mb and was chosen manually (Fan and Gijbels, 1996).

### Map and Genetic Diversity of Chromosome 4A

A table (Supplementary Table S4 in Akhunov et al., 2010) containing SNP diversity statistics for expressed sequence tag (EST)-derived sequences in 10 accessions of wild emmer from the Karaca Dağ region and 13 accessions of *T. aestivum* ssp. *aestivum*, *compactum*, and *spelta*, was downloaded. The starting nucleotides on the *A. tauschii* pseudomolecules (GenBank BioProject PRJNA341983) for ESTs for which diversity data existed in the table were determined and the locations of the ESTs on the genetic map were imputed using the locations of the 90K wheat iSelect Infinium markers on the *A. tauschii* pseudomolecules as references. Diversity statistics, Watterson nucleotide polymorphism estimator θ*w* (Watterson, 1975), nucleotide diversity θπ (Nei and Li, 1979), and Tajima’s *D* (Tajima, 1989) were computed (Akhunov et al., 2010) and θπ was graphed to assess diversity distribution along the genetic map of chromosome 4A in Karaca Dağ wild emmer and *T. aestivum*.

## Results

### Genetic Map Construction

DNAs from the 445 independent F_6_ to F_8_ RILs from the cross Landon x PI 428082 were genotyped with the 90K wheat SNP iSelect Infinium assay. Sixteen (3.7%) RILs were removed from the population because of various genotyping defects, leaving 429 RILs for further analyses. The 90K Infinium assay contained 26,385 D-genome markers and 55,038 A- and B-genome markers. Only the latter were *a priori* relevant to tetraploid wheat genotyping. Of these, 13,422 (24.1%) markers were polymorphic between Langdon and PI 428082 and generated well-clustered genotyping graphs with GenomeStudio. A small portion, 138 (0.5%), of the markers classified as D-genome markers in the 90K Infinium database (Wang et al., 2014) also generated well-clustered SNP genotyping graphs.

In total, 13,560 markers produced well-clustered genotype data and were used in map construction. After two cycles and manual editing of data for spurious genotype calls, a map consisting of 10,914 markers was produced (**Table 1**). The map was comprised of 2,650 segregating markers (referred in Multipoint ULD program as skeleton markers), which were single markers representative of a bin of co-segregating markers (Supplementary Table S1) and 8,264 co-segregating markers (referred in Multipoint ULD program as bound markers) (Supplementary Table S2).

**Table 1:** Characteristics of the 16 linkage groups including separate linkage groups for the four segments involved in the 3B-6B reciprocal translocation.

Linkage group	Length (cM)	Segregating markers (no.)	Co-segregating markers (no.)	Total segregating and co- segregating markers (no.)	Markers collinear with the *A. tauschii* pseudomolecules (no.)
1A	112.76	188	490	678	604
1B	116.27	217	694	911	763
2A	124.8	168	551	719	625
2B	122.86	236	1110	1346	1173
3A	133.83	220	533	753	619
3BS	47.55	84	270	354	256
3BL	68.69	125	354	479	399
4A	128.19	163	390	553	456
4B	108.03	141	365	506	364
5A	176.48	177	445	622	482
5B	138.66	229	755	984	871
6A	101.55	155	394	549	499
6BS	46.82	71	247	318	253
6BL	48.94	82	404	486	391
7A	135.1	221	576	797	635
7B	117.38	173	686	859	653
Total	1727.93	2,650	8,264	10,914	9,043

Fourteen LGs were obtained. Twelve were consistent with the wheat 90K Infinium consensus map (Wang et al., 2014), one was chimeric and consisted of arms 3BL, 6BS, and 6BL, and one contained arm 3BS. The latter LG merged with the chimeric LG when the requirement RF < 0.1 was relaxed. The linkage between proximal markers in 3BS and 6BL and 6BS and 3BL indicated that the chimeric LG was caused by a 3B-6B reciprocal translocation in wild emmer with breakpoints in the 3B and 6B centromeric regions. The four chromosome arms making up the 3B-6B reciprocal translocation were purposefully kept as separate LGs throughout the work reported here even though 3BS and 6BL LGs and 6BS and 3BL LGs were linked across the centromeres. The resulting 16 LGs (Supplementary Figure S1) had a total length of 1,727.93 cM and had an average of one segregating marker every 0.65 cM. The lengths of the LGs of chromosomes that were not involved in the 3B-6B translocation ranged from 101.55 cM for 6A to 176.48 cM for 5A (**Table 1**).

For downstream applications, it was desirable to use all markers present on the map, not just the segregating markers. To order the 8,264 co-segregating (bound) markers that co-segregated with the segregating (skeleton) markers, the sequences of all 10,914 markers were used as queries in BLASTN homology searches against the *A. tauschii* pseudomolecules (GenBank BioProject PRJNA341983) and hits with expect value < E-5 were recorded (Supplementary Table S3). The segregating markers were then arranged according to their locations in the LGs whereas co-segregating markers in each co-segregating bin were ordered according to their starting nucleotides on the *A. tauschii* pseudomolecules so that their ascending or descending progression was consistent with the progression of the neighboring segregating markers (Supplementary Table S3). Of the 10,914 markers, 9,131 (83.6%) were ordered using this strategy (**Table 1** and Supplementary Table S3).

Some markers hit many sites on the *A. tauschii* pseudomolecules in BLASTN searches (Supplementary Table S4), suggesting that they may have been derived from repeated sequences. The highest number of hits was 7,165 for marker Tdurum_contig28050_299. We chose > 10 hits in the *A. tauschii* pseudomolecules as an arbitrary threshold for considering a SNP marker to be derived from a repeated sequence. Using this threshold, 42 (0.4%) of the 10,914 SNP markers were derived from repeated sequences (Supplementary Table S4).

This expanded genetic map and marker locations on the *A. tauschii* pseudomolecules were used to compute recombination rates along the 14 chromosomes, which were expressed as cM per Mb (Supplementary Figure S2).

### Chromosome Rearrangements

Disregarding temporarily the previously known structural rearrangements involving chromosomes 4A, 5A, and 7B, 15 structural rearrangements relative to the order of markers along the *A. tauschii* pseudomolecules were found (**Table 2**). Recombination was detected within all rearrangements suggesting that, except for the 3B-6B translocation, all were likely shared by the parents and were homozygous in the F_1_ generation.

**Table 2:** Summary of structural rearrangements relative to the order of markers along the *A. tauschii* pseudomolecules, and the presence of these rearrangements on the consensus genetic maps of Wang et al. (2014) and Maccaferri et al. (2015).

Rearrangement	Chromosome arm	SNP marker	cM	Length (bp)	Traversed distance (bp)	Present in Maccaferri et al. (2015)	Present in Wang et al. (2014)
Intrachromosomal translocation	1AL	wsnp_BE585780A_Ta_2_1	37.77	6,145,185	1,546,021	Yes	
		Tdurum_contig81558_113	37.77				
Intrachromosomal translocation	1AL	BS00022261_51	62.94	381,169	776,520	Yes	
		GENE-0262_431	63.05				
Intrachromosomal translocation	1BS	GENE-0427_115	29.16	1,565,889	2,092,637	No	Yes
		Kukri_c60285_505	29.16				
Intrachromosomal translocation	1BL	Tdurum_contig13405_483	35.28	3,165,279	4,200,142	No	Yes
		IAAV6919	35.76				
Intrachromosomal translocation	2AS	BobWhite_c2532_966	62.11	6,203,600	6,640,539	Yes	
		RAC875_rep_c74200_1415	62.11				
Intrachromosomal translocation	3AS	Kukri_rep_c96809_352	4.26	532,098	903,789	Yes	
		wsnp_Ex_c2573_4788116	4.26				
Intrachromosomal translocation	3AS	Excalibur_c74666_291	4.50	384,672	1,774,336	Yes	
		BobWhite_c23392_496	4.50				
Intrachromosomal translocation	6AS	D_GB5Y7FA01EBNCV_218	35.60	3,353,890	4,963,447	No	Yes
		BobWhite_c5782_825	35.71				
Intrachromosomal translocation	7BS	Tdurum_contig68742_597	46.06	4,668,693	14,089,439	Yes	
		BS00095819_51	46.06				
Interchromosomal translocation	7BL	Excalibur_rep_c69070_180	53.53	3,369,543	63,280,261	Yes	
		wsnp_Ex_c4213_7609689	53.53				
Paracentric inversion Inv(1)	3AS	BS00022798_51	3.90	2,282,223	NA	Yes	Yes
		Excalibur_c74666_291	4.50				
Paracentric inversion Inv(2)	7AL	wsnp_Ex_c29371_38412298	69.73	26,801,048	NA	Yes	
		wsnp_Ex_c558_1105911	70.44				
Paracentric inversion Inv(3)	7AL	BS00048915_51	70.80	27,053,119	NA	Yes	
		BS00065250_51	72.83				
Paracentric inversion Inv(4)	7BS	BS00022562_51	0.00	414,765	NA	Yes	
		Excalibur_c37696_192	0.23				
Reciprocal translocation	3B centromere	TA004110-0731	47.55	NA	1,392,493	No	No
	6B centromere	RAC875_c77_1176	46.82				

The most frequent type of chromosome rearrangement was an intrachromasomal translocation. Nine were detected and all were short, ranging in length from 134,271 to 6,145,185 bp as measured on *A. tauschii* pseudomolecules, and in all of them a chromosome segment was translocated only a short distance (**Table 2**). Our map shared most of the intrachromosomal translocations with the durum consensus map (Maccaferri et al., 2015) or common wheat consensus map (Wang et al., 2014) (**Table 2**).

The second most frequent type of chromosome rearrangement was a paracentric inversion. Three were large, Inv(1) detected in arm 3AS, Inv(2) detected in arm 7AL, and Inv(3) juxtaposed in arm 7AL to Inv(2) (**Table 2**). The fourth inversion, Inv(4), was only 0.23 cM long, and was at the tip of arm 7BS, in the 5AL segment translocated to 7BS. Our map shared the order of markers in these four inversions with the durum consensus map (**Table 2**), and in Inv(1), also with that on the consensus map of common wheat (**Table 2**).

There were two interchromosomal translocations. One was between 6BS and 7BL. The segment present in 7BL was absent on the 6BS genetic map, suggesting that the rearrangement was non-reciprocal. The translocation was also present on the durum consensus map (Maccaferri et al., 2015) (**Table 2**). The other interchromosomal translocation was the reciprocal translocation 3B-6B described above. No segmental duplication satisfying our arbitrary requirement to involve three consecutive genes was detected on the genetic map.

### Ancestral and Derived States of Marker Order in Inversions

The marker progressions within the inverted and flanking regions in LGs 3A, 3B, 7A, 7B, and the homoeologous *A. tauschii, B. distachyon*, rice, and sorghum pseudomolecules were determined (Supplementary Table S5) to distinguish between the ancestral and derived states of marker order for these specific rearrangements. For Inv(1), LGs 3A and 3B and the Bd2, Os1, and Sb3 pseudomolecules had the same marker order whereas pseudomolecule 3D had the alternative order (Supplementary Table S5). The fact that the outgroup *B. distachyon*, rice, and sorghum pseudomolecules had the same marker order as LG3A and 3B indicated that this order was the ancestral state and that of 3D was a derived (inverted) state.

No markers were mapped on the 7B map in the region corresponding to Inv(2) and the proximal part of Inv(3). Therefore, only a distal portion of Inv(3) was studied in colinearity comparison including homoeologous regions of LG7A and 7B and pseudomolecules 7D, Bd1, Os6, and Sb10. Marker order was shared by LG7A and Bd1, Os6, and Sb10 whereas LG7B and the 7D pseudomolecule shared the alternative order (Supplementary Table S5). Following the same rationale as above, we concluded that the 7A marker order was the ancestral state and that in LG7B and pseudomolecule 7D was the derived (inverted) state.

### 4A-5A-7B Structural Rearrangements

**FIGURE 1:**
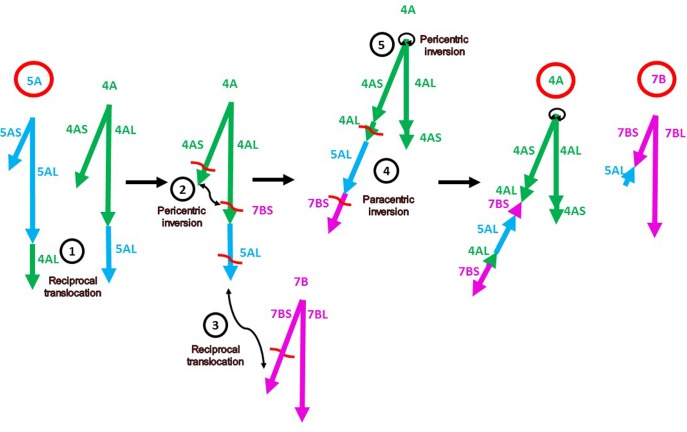
Diagram of the current model (Devos et al., 1995; Mickelson-Young et al., 1995; Miftahudin et al., 2004) of evolution of the present-day rearranged wheat chromosomes 4A, 5A, and 7B (labeled with bold red circles). The chromosome arms of the ancestral chromosomes 4A, 5A, and 7B are indicated by green, blue, and magenta arrows, respectively (as labeled in Supplementary Table S3). The directions of the arrows indicate the gene order in the centromere-telomere direction on the corresponding *A. tauschii* pseudomolecules. The numbers in the black circles correspond to the events described and enumerated in Results. The chromosome arm designations refer to the ancestral chromosomes. The red wavy lines are hypothetical breakpoints.

The alignment of the chromosome 4A, 5A, and 7B markers on the *A. tauschii* pseudomolecules confirmed the locations and orientations of major segments previously reported in these rearranged chromosomes (Devos et al., 1995; Mickelson-Young et al., 1995; Nelson et al., 1995; Miftahudin et al., 2004; Hernandez et al., 2012). The following complex sequence of events leading to the evolution of the rearranged chromosomes 4A-5A-7B has been proposed (**Figure 1**) (Devos et al., 1995; Mickelson-Young et al., 1995; Miftahudin et al., 2004): (1) A reciprocal 4AL-5AL translocation, which exchanged the distal portions of 4AL and 5AL arms. This translocation originated in the diploid ancestor of the wheat A genome. (2) A pericentric inversion in 4A, which converted the short arm to the present-day long arm and a remnant of the long arm became the present-day short arm. (3) A reciprocal translocation between 4AL (originally 5AL) and 7BS. (4) A paracentric inversion that inverted the 4AL and 5AL segments in the 4AL arm. (5) A small 4A pericentric inversion. Our genetic map in concert with the *A. tauschii* reference sequence confirmed rearrangements (1), (3), and (5) but revealed the following discrepancies.

We obtained an unequivocal evidence for the presence of genes of the ancient 4AL at the end of the ancient 4AS (present-day 4AL) but could not confirm the presence of two EST markers (BE518074 and BE494743) in the terminal region of the present-day arm 4AS (Supplementary Table S6). No other short arm marker from this region was mapped distal to the ancient 4AL fragment making up the present-day 4AS (Supplementary Table S3). We therefore failed to obtain evidence for the second breakpoint of the hypothetical large pericentric inversion.

The breakpoints of the distal paracentric inversion (4) in the present-day 4AL were proposed to be in the short 4AL segment and the 7BS segment (**Figure 1**). If that were true, a portion of the 7BS segment should have been moved to a proximal position relative to the 5AL segment and a portion of the 4AL segment should have remained proximal to the 5AL segment, as shown in **Figure 1**. Proximal locations relative to the 5AL segment of two 4AL ESTs (BE499664 and BE637934) and four 7BS ESTs (Supplementary Table S6) were used as supporting evidence for these breakpoints (Miftahudin et al., 2004). However, none of the six ESTs were proximal to the 5AL segment on our genetic map and genes homologous to them were in different locations in the *A. tauschii* reference genome sequence (Supplementary Table S6). The ancient 4AL, 5AL, and 7BS segments making up the distal portion of the present-day 4AL were intact on our genetic map and showed no signs of breakpoints causing the paracentric inversion (4).

### Evolution of 4A

The short arm of the present-day chromosome 4A comprises only 76% of the proximal portion of the ancient 4AL chromosome arm. Terminal deletions of this size greatly reduce or entirely preclude meiotic pairing with the wild-type homologs (Curtis et al., 1991). The long arm of the rearranged wheat chromosome 4A is composed of four segments. Of these, only the terminal 7BS segment shares a telomere and orientation with the ancient chromosome 7B and could have paired and recombined with it (**Figure 1**). The remaining segments are in different positions and inverted orientations compared to the ancient wild-type chromosomes (**Figure 1**). As a result, the rearranged wheat chromosome 4A could not have paired with the wild-type chromosomes, except for the distal 7BS segment, and except for this segment, the rearrangements would have severely limited gene flow into the rearranged chromosome 4A.

Selection for the rearranged chromosome 4A would have led to a selective sweep involving most of the chromosome, except for the terminal 7BS segment. That being the case, most if not all diversity that exists in the rearranged chromosome 4A, except for the 7BS segment, must have originated since the evolution of the present-day chromosome 4A. Hence, nucleotide diversity of wheat 4A relative to that in the rest of the A-genome can be used to assess the relative age of the rearranged wheat chromosome 4A. The more recent are the rearrangements, the less diverse the chromosome will be relative to the remaining six A-genome chromosomes.

**FIGURE 2:**
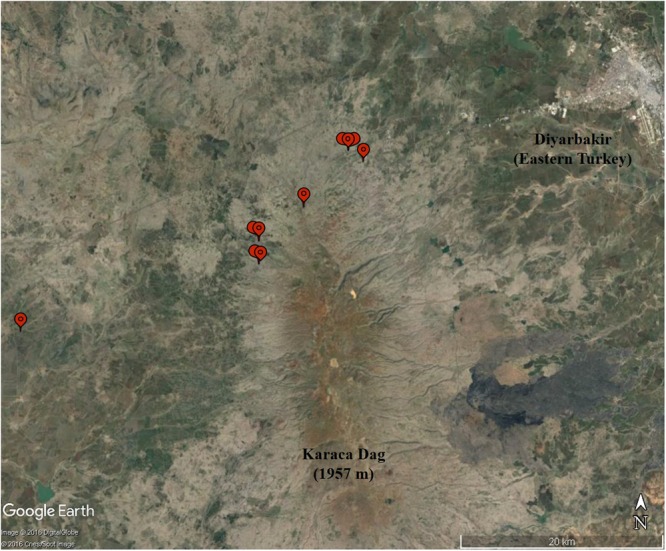
The collection sites (balloons) of the 10 wild emmer accessions in the Karaca Dağ region. Note the proximity of the region to Diyarbakir in the upper right corner.

Akhunov et al. (2010) reported nucleotide diversity at 2,114 expressed sequence loci based on Sanger sequencing in 12 accessions representative of *T. aestivum* and 10 accessions of wild emmer from the Karaca Dağ region (**Figure 2**). The data included 110 4A loci. Nucleotide diversity in 4A was significantly lower than the A-genome population mean (Akhunov et al., 2010) but without a genetic map, the distribution of genetic diversity along the rearranged chromosome 4A could not have been fully interpreted (Akhunov et al., 2010).

We used our genetic map of 4A in re-analyzing these data by re-computing the mean Watterson nucleotide polymorphism measure θ*w*, nucleotide diversity θπ, and Tajima’s *D* for six A-genome chromosomes without 4A and for 4A itself (**Table 3**). In *T. aestivum*, θ*w* but not θπ was significantly lower in 4A than in the remaining six A-genome chromosomes (**Table 3**). In wild emmer from the Karaca Dağ region, both diversity measures were significantly lower in 4A than in the remaining A-genome chromosomes (**Table 3**).

**Table 3:** Average Waterson nucleotide polymorphism (θw), nucleotide diversity (θπ), and Tajima’s D among 12 accessions representative of *T. aestivum* and 10 accessions of wild emmer collected in the Karaca Dağ region in Turkey.

Chromosome or chromosome region	θ*w* × 10^-3^		θπ × 10^-3^		Tajima’s *D*	
	*T. aestivum*	Wild emmer	*T. aestivum*	Wild emmer	*T. aestivum*	Wild emmer
A genome^$^	0.62	0.68	0.60	0.73	-0.07	0.18


4A^$$^	0.45^∗^	0.59	0.42^∗^	0.50^∗^	-0.11	-0.39


4AS distal^#^	0.38^∗^	0.57	0.27^∗^	0.51	-0.62	-0.28


Sweep area^#^	0.47	0.03^∗^	0.65	0.01^∗^	0.95	-1.11^∗^


4AL distal^#^	0.68	1.10^∗^	0.44	0.96^∗^	-0.71	-0.33

^$^*Recomputed from Table 8 in Akhunov et al. (2010) by excluding 4A. ^$$^From Akhunov et al. (2010). ^#^Diverse, distal portions of chromosome 4A short and long arms and the proximal region including the selective sweep in wild emmer as delimited in wild emmer (Supplementary Table S7). ^∗^Means outside 99% bootstrap significance interval of the genome mean*.

**FIGURE 3:**
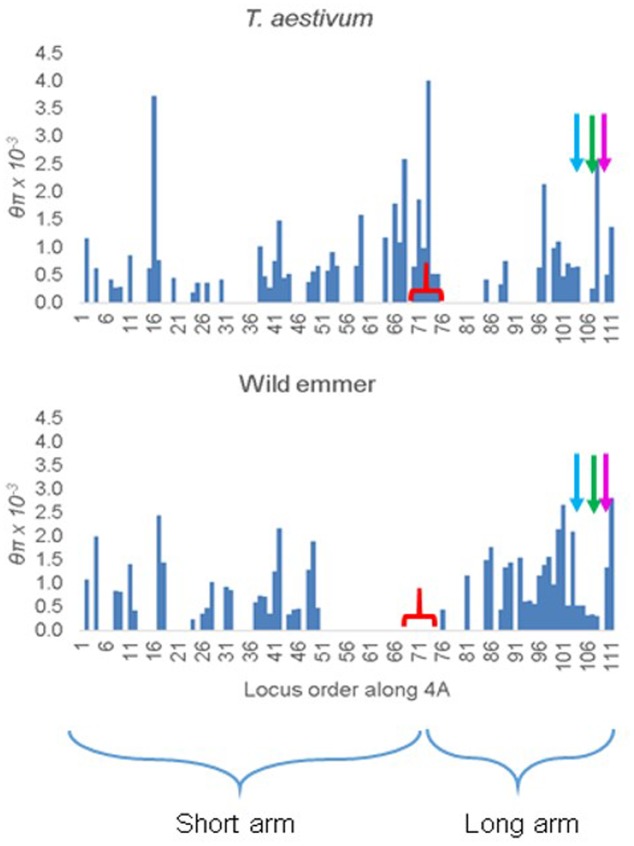
Distribution of nucleotide diversity θπ along chromosome 4A in 12 representative accessions of *T. aestivum* and 10 accessions of wild emmer from the Karaca Dağ region. The markers are ordered according to their order along the 4A genetic map. The tip of the short arm is to the left. The starts of the ancient 5A segment (blue arrow), ancient 4AL segment (green arrow), and the ancient 7BS segment (magenta arrow) in the 4AL arm are indicated. The approximate location of the centromere is indicated by a red bracket.

In *T. aestivum*, θπ was uniformly high along 4A (**Figure 3**). For an unknown reason, loci with diversity estimates reported by Akhunov and his colleagues were disproportionally under-represented in the long arm of 4A, particularly in the 5AL, 4AL, and 7BS segments (**Figure 3**). Nevertheless, in the few loci that were investigated in these segments nucleotide diversity was similar to that in the rest of the chromosome.

In Karaca Dağ wild emmer, the centromeric region involving 30 loci showed very low diversity indicating a selective sweep (**Figure 3** and Supplementary Table S7). While θπ was 0.51 × 10^-3^ and 0.96 × 10^-3^ in the distal regions of wild emmer 4A (**Table 3**), θπ was only 0.01 × 10^-3^ in the centromeric region, although θπ was 0.65 × 10^-3^ in the same region in *T. aestivum.* In the sweep area, Tajima’s *D* was highly negative in wild emmer (**Table 3**), which is indicative of recent selective sweep followed by population expansion.

## Discussion

### Maps

Of the 90K Infinium SNP markers 13,560 were polymorphic between Langdon and wild emmer accession PI 428082 and 2,650 were mapped with the population of 429 RILs as segregating (skeleton) markers. For comparison, 16,387 90K Infinium markers were polymorphic in a mapping population of 150 F_6_ RILs from a cross between durum ‘Svevo’ and wild emmer ‘Zavitan’ from northern Israel (Avni et al., 2014) but only 2,297 were mapped as segregating (skeleton) markers (Avni et al., 2014). This difference in mapping outcomes highlights the importance of the number of RILs in the mapping population for mapping efficiency.

Recombination rates were high in the distal regions of chromosomes and declined toward the proximal regions. This pattern is consistent with other recombination rate studies in wheat and its close relatives in the tribe Triticeae (Dvorak and Chen, 1984; Lukaszewski and Curtis, 1993; Dubcovsky et al., 1996; Gill et al., 1996; Zhang et al., 2001; Akhunov et al., 2003; Luo et al., 2005, 2013; Avni et al., 2014). In the Langdon x PI 428082 population, the recombination rates declined more precipitously in the short arms than in the long arms. In the long arms of chromosomes 1, 4, and 5 and the arms of large metacentric chromosomes 2, 3, and 7, the rates peaked about 50 Mb from chromosome termini (Supplementary Figure S2).

An important factor affecting recombination in wheat is the homoeologous pairing suppressor *Ph1*. The suppressor acts on polymorphism between recombining chromosomes (Luo et al., 1996). The greater is polymorphism, the greater is recombination rate suppression. This inverse relationship accounts for the short lengths of LGs observed on our map, particularly in the B genome chromosomes which, for an unknown reason, are affected by polymorphism more than the A-genome chromosomes (Dvorak and McGuire, 1981). While the average A-genome LG was 130.3 cM, the average B-genome LG was only 116.4 cM (*P* = 0.03, paired *t*-test).

A factor that undoubtedly confounded estimation of recombination rate in the B genome was heterozygosity for the 3B-6B reciprocal translocation. Reciprocal translocations are common in wild emmer and are more frequent in the B-genome than in the A-genome (Kawahara, 1986, 1987). We do not know the frequency of the 3B-6B translocation described here in the wild emmer population, because accession PI 428082 was not included in the Kawahara’s study. Heterozygosity for a reciprocal translocation reduces recombination rates in the chromosome arm that includes a break (Dobzhansky, 1931). The total length of chromosome 3B (sum of the 3BS and 3BL LGs) was 116.2 cM and total length of chromosome 6B (sum of the 6BS and 6BL LGs) was 95.8 cM. Both chromosomes were shorter than the mean genetic length of the remaining five B-genome chromosomes, 120.6 cM. The short length of the two chromosomes was particularly notable for chromosome 3B which is physically the largest wheat chromosome (Dvorak et al., 1984). Both 3B and 6B were genetically also shorter than their A-genome homoeologs.

### Structural Chromosome Evolution

The comparison of the genetic map with the *A. tauschii* reference genome sequence uncovered 15 chromosome rearrangements, in addition to the known 4A-5A-7B rearrangements. Four of these were paracentric inversions, Inv(1) to Inv(4). Since inversion heterozygosity suppresses recombination in the inverted region (except for two-strand double crossovers), high levels of recombination within the inverted regions in the Langdon x PI 428082 F_1_ indicate that the F_1_ plants were homozygous for these inversions and that these inversions are shared by Langdon and PI 428082. Moreover, all four inversions were present on the durum consensus map (Maccaferri et al., 2015) suggesting that they are widely distributed in wild and domesticated tetraploid wheat.

**FIGURE 4:**
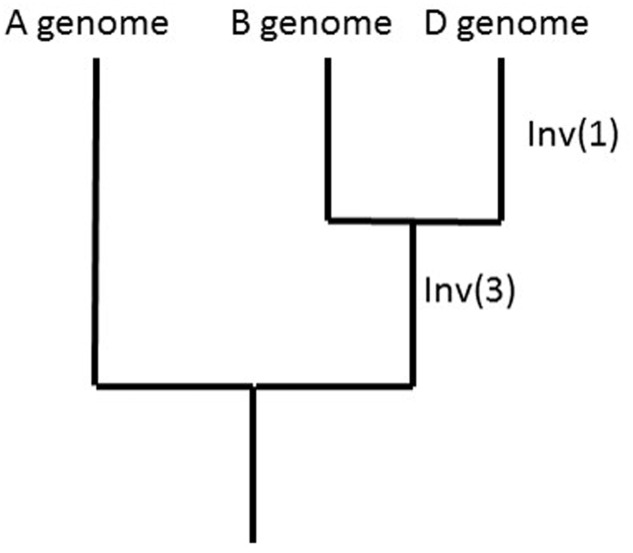
Phylogeny of the A, B, and D genomes as suggested by the sharing of Inv(1) and Inv(3) among the A, B, and D genomes and outgroup genomes of *Brachypodium distachyon*, rice, and sorghum.

Comparisons of the A-genome and B-genome LGs with the *A. tauschii, B. distachyon*, rice, and sorghum pseudomolecules showed for Inv(1) that the derived (inverted) state is found in 3D. A similar analysis showed for Inv(3) that the derived (inverted) state is shared by 7B and 7D. The ancestral vs. derived state of Inv(2) could not be investigated. Because the likelihood of a reversion of an inverted segment is small, sharing of inversions among genomes can be used to reconstruct phylogeny, as shown for the A, B, and D genomes (**Figure 4**).

Nine of the rearrangements were intrachromosomal translocations. In all of them, the translocated segment was short and was translocated only a short distance along the chromosome. It is tempting to attribute these translocations to transposition, since transposable elements (TEs) tend to transpose short distances. However, the same outcome could be produced by intrachromosomal crossover between TEs, which excises a circular intermediate that can be reinserted in the vicinity. It is therefore impossible to say without more analytical work whether the intrachromosomal translocations originated via transposition or ectopic recombination.

### Wheat Chromosome 4A

Several attempts based on RFLP and deletion maps have been made to reconstruct the evolution of the rearranged wheat chromosome 4A (Devos et al., 1995; Mickelson-Young et al., 1995; Miftahudin et al., 2004). Our wild emmer genetic map failed to confirm the current models of evolution of this chromosome. Specifically, it failed to validate one breakpoint of the pericentric inversion (2) and both breakpoints of the paracentric inversion (4) previously reported based on EST locations on the 4A maps. We failed to find the 7BS EST loci proximal to the 5AL segment and confirmed thus a similar failure to locate these EST loci in the survey sequence of 4A (Hernandez et al., 2012). A radiation hybrid map of 4A revealed discrepancies in the 4AL deletion breakpoints (Balcárková et al., 2017), which may account for the conflicting interpretation of EST locations. Clearly, the structure of chromosome 4A needs reassessment but we prefer the revisit it after a reference-quality sequence of wheat is available.

### Timing of Evolution of the Rearranged 4A

There are two sources of genetic diversity (θπ) in a polyploid species: introgression from the diploid progenitors (θπ_i_) and mutations that have occurred since the origin of the polyploid species (θπ_p_). Total diversity θπ of a polyploid is therefore θπ_i_ + θπ_p._ The suppression of recombination and fixation of the rearranged chromosome 4A in wild emmer swept away θπ_i_ from the majority of 4A genes, and θπ_i_ can be assumed to be zero. The magnitude of θπ_p_ in 4A and θπ_p_ in the remaining A-genome chromosomes can therefore be used to estimate the age of the rearranged 4A chromosome relative to the age of wild emmer, provided that θπ_i_ in the remaining chromosomes can be estimated and subtracted from total diversity θπ. Wheat D genome is less than 8,000 years old, and most of its diversity, θπ = 0.18 × 10^-3^, was contributed by gene flow from *A. tauschii* (Akhunov et al., 2010; Wang et al., 2013). We will therefore use the diversity in the wheat D genome as an estimate of θπ_i_. Subtracting 0.18 × 10^-3^ from the total diversity in the *T. aestivum* non-4A A-genome chromosomes estimates θπ_p_ = 0.42 × 10^-3^. Remarkably, this estimate is identical to the estimate of total diversity θπ = 0.42 × 10^-3^ in the 4A of *T. aestivum* (**Table 3**). The same manipulation estimates θπ_p_ = 0.55 × 10^-3^ (0.73 - 0.18 × 10^-3^) in the wild emmer non-4A A-genome chromosomes. This estimate is close to total diversity θπ = 0.50 × 10^-3^ in wild emmer 4A.

We can take into consideration the diversity sweep in the wild emmer 4A and exclude that from the comparison. Then θπ_p_ = 0.51 × 10^-3^ in the short arm of 4A and 0.96 × 10^-3^ in the long arm of 4A (**Table 3**), making the agreement between total diversity in 4A and θπ_p_ in the remaining six A-genome chromosomes slightly closer.

It might be of interest to compare 4A diversity with diversity in the 5AL and 7BS segments involved in the 4A-5A-7B translocation. Unfortunately, diversity of only two genes in the 5AL segment and one gene in the 7BS segment was reported (Akhunov et al., 2010), which is inadequate for a meaningful comparison.

Both in *T. aestivum* and wild emmer, the levels of total diversity θπ in 4A are similar to θπ_p_ in the remaining six A-genome chromosomes. Assuming that diversity has been generated with equal rates in all A-genome chromosomes, these diversity levels suggest that the fixation of the rearranged chromosome 4A and the origin of wild emmer may have been contemporary or the rearrangements took place very early in the evolution of wild emmer.

### Role of Karaca Dağ Wild Emmer in Emmer Domestication

The Karaca Dağ region includes several archeological sites on the upper Euphrates and Tigris rivers with some of the oldest records of agriculture, and this area is viewed by some as the cradle of agriculture in western Asia (Lev-Yadun et al., 2000). Evidence for domestication of emmer in the Karaca Dağ region (Nelson et al., 1995; Ozkan et al., 2002, 2005, 2011; Luo et al., 2007) is critical for this hypothesis.

If emmer were indeed domesticated in the Karaca Dağ region, wild emmer in the Karaca Dağ region would be the ancestor of all domesticated tetraploid and hexaploid wheat. Yet, evidence for gene flow between domesticated emmer and wild emmer in all areas where the two have been sympatric (Luo et al., 2007) raises concerns about the purity of wild emmer in general and in Karaca Dağ region in particular.

Another complicating factor is the selective sweep apparent in the centromeric region of chromosome 4A of wild emmer in the Karaca Dağ region. This sweep was previously detected with 10 RFLP loci in the centromeric region of 4A in a sample of 48 accessions from the Karaca Dağ region and 117 accessions of the northern population of domesticated emmer (Dvorak et al., 2006a). This sweep is perplexing in light of the fact that the same chromosome region in *T. aestivum* and wild emmer in other geographic regions (Avni et al., 2017) show normal levels of nucleotide diversity. If wild emmer in the Karaca Dağ region were ancestral to all domesticated wheat, what was the source of the diversity in *T. aestivum*? In addition, what is the cause of the selective sweep in the Karaca Dağ wild emmer population?

Diversity of the *Psr920-4A* RFLP locus (= ABCT-1 gene) suggests a possible answer to the first question. The locus is in the centromeric region of 4A and is dimorphic. With few rare exceptions, wild emmer from all areas of the Fertile Crescent has the *Psr920b* allele whereas all domesticated tetraploid wheats have the *Psr920a* allele (Dvorak et al., 2006a). Hexaploid wheat has both alleles (Dvorak et al., 2006a) suggesting introgression from wild emmer into chromosome 4A of *T. aestivum*. Thus, gene flow from wild emmer in regions outside Karaca Dağ could have possibly contributed diversity in the sweep area in *T. aestivum*.

We can provide no satisfactory explanation for the second question. A number of causes are possible, such as selection for adaptation to the Karaca Dağ environment or selection favoring a wild allele at a domestication gene on 4A. Since a similar selective sweep appears to exist also in the northern population of domesticated emmer, we cannot rule out even the extreme scenario that wild emmer in the Karaca Dağ region is actually feral (Civan et al., 2013) and thus derived from the northern population of domesticated emmer. These possibilities call for renewed attention to the purity of the Karaca Dağ wild emmer population and to the genetic relationships between it and other populations of wild and domesticated tetraploid wheat. The population of RILs developed here may be instrumental for mapping domestication genes in Karaca Dağ wild emmer and shedding light on this dilemma.

## Author Contributions

CJ and JD planned the study. CJ conducted most of the experimental work with assistance and advice from AD. BG generated the genetic diversity data. JD, CJ, RR, M-CL, and JD with assistance from AK and AD analyzed data. CJ and JD wrote the first draft of the paper and all authors assisted with the development of the final draft.

## Conflict of Interest Statement

The authors declare that the research was conducted in the absence of any commercial or financial relationships that could be construed as a potential conflict of interest.
