# Supplementary figures and images for: A High-Density Genetic Map of Wild Emmer Wheat from the Karaca Dağ Region Provides New Evidence on the Structure and Evolution of Wheat Chromosomes
Source: Front Plant Sci. 2017 Oct 20;8:1798. doi: 10.3389/fpls.2017.01798 (PMC5655018; doi:10.3389/fpls.2017.01798)

Figure S1

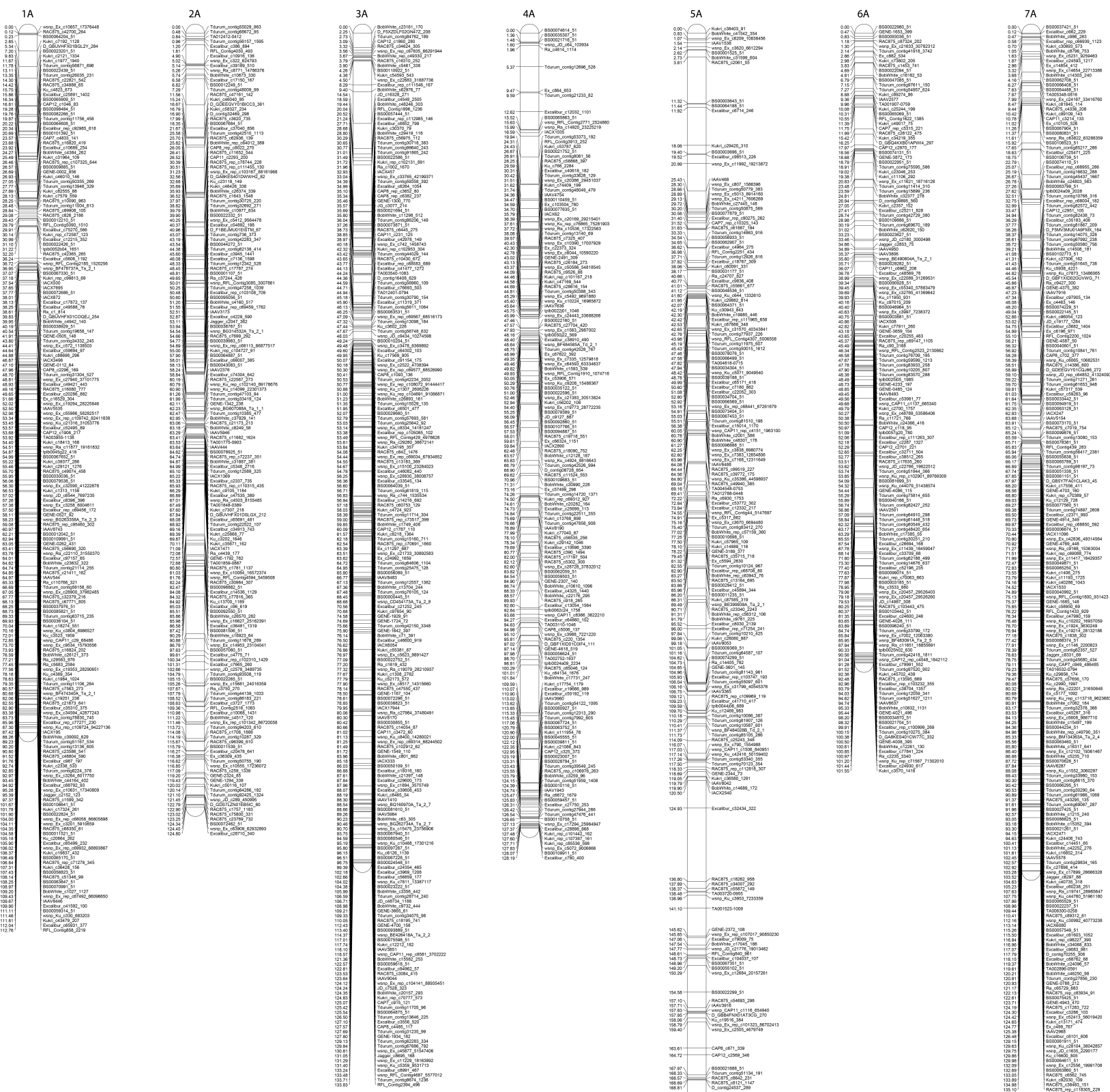

Figure S1

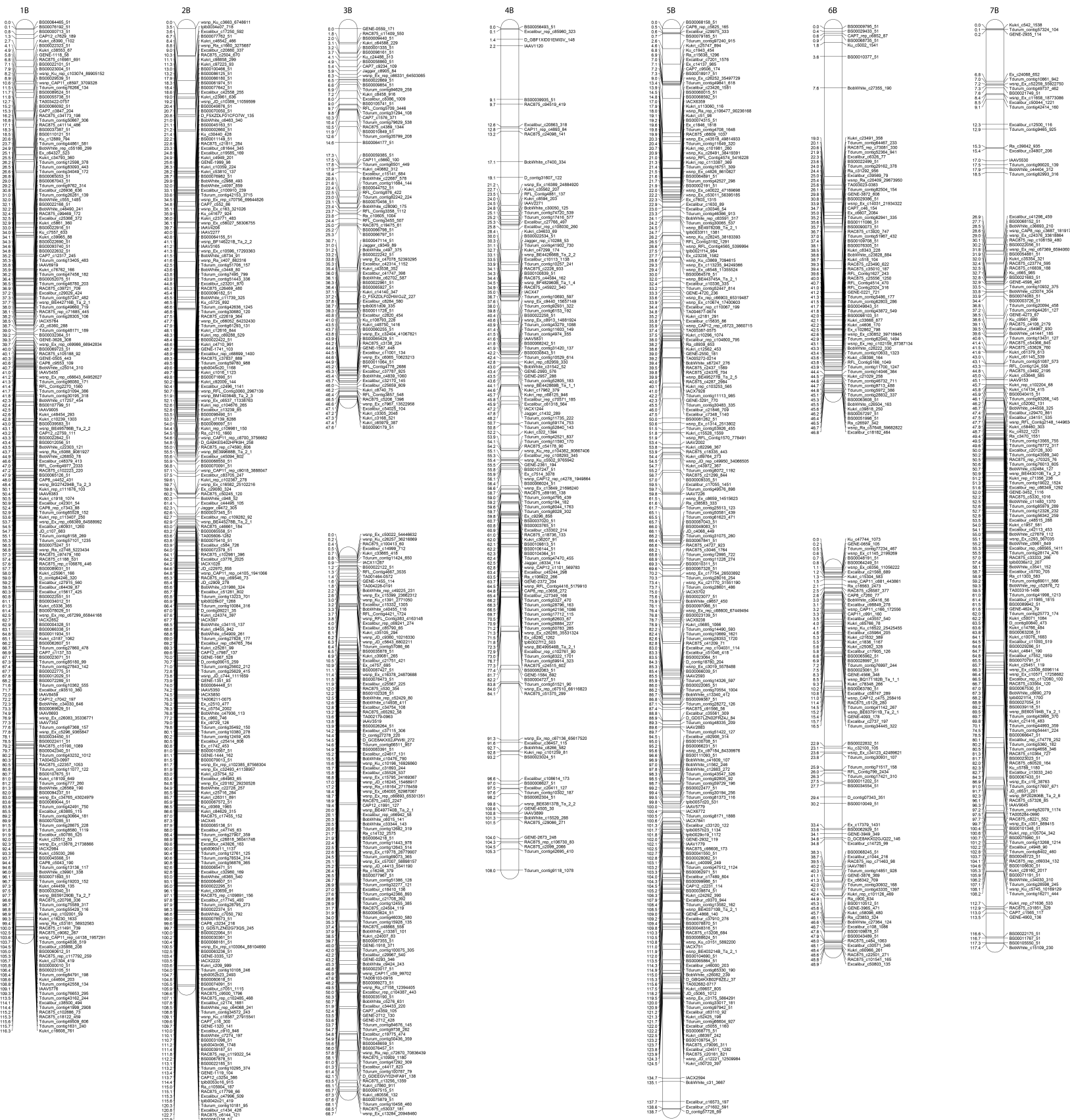

Supplement: Supplementary file 8 [file Image_1.pdf]

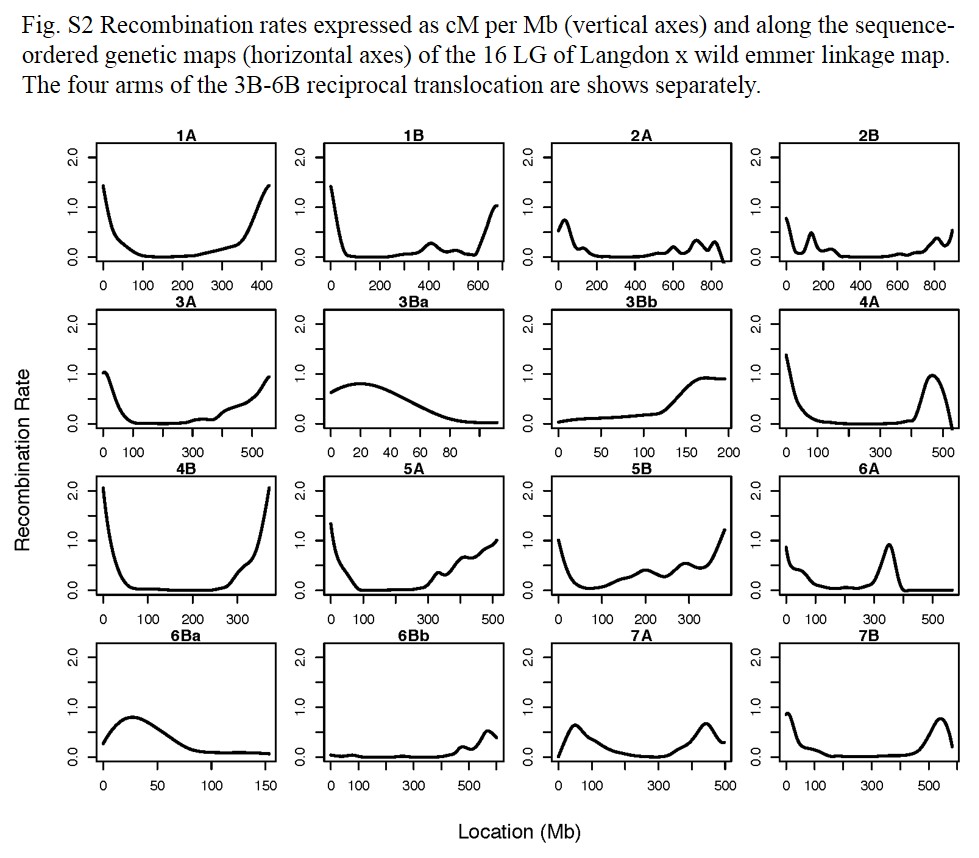

Supplement: Supplementary file 9 [file Image_2.JPEG]
